# Effectiveness of perampanel in the treatment of pediatric patients with focal epilepsy and ESES: A single-center retrospective study

**DOI:** 10.3389/fphar.2022.1026836

**Published:** 2022-10-07

**Authors:** Tao Yu, Zi-Teng Teng, Xue-Yan Liu, Hua Wang

**Affiliations:** Department of Pediatrics, Shengjing Hospital of China Medical University, Shenyang, Liaoning, China

**Keywords:** epilepsy, ESES, SWI, perampanel, pediatric patients

## Abstract

**Objective:** To investigate the therapeutic effect and influencing factors of perampanel (PER) on electrical status epilepticus during sleep (ESES).

**Methods:** We retrospectively analyzed the clinical data of pediatric patients with focal epilepsy and ESES who were treated at the Epilepsy Center of Shengjing Hospital of China Medical University between January 2016 and March 2022. Changes in the spike wave index (SWI) after 24 weeks of PER add-on treatment were compared. Kaplan‒Meier survival analysis, the log-rank test and multivariate Cox regression analysis were performed.

**Results:** A total of 54 pediatric patients met the inclusion criteria, including 33 males and 21 females. The mean age at the diagnosis of epilepsy was 6.41 ± 2.14 years and at ESES diagnosis was 7.58 ± 2.40 years. The mean ESES duration before add-on PER was 25.31 ± 15.12 months. The mean age of the patients at add-on PER initiation was 9.69 ± 2.12 years. The ESES resolved in 29 children after 6 months of PER add-on treatment, and the response rate was 53.7%. Univariate analysis with the log-rank test showed that the therapeutic effect of PER differed according to the age at ESES diagnosis and ESES duration before add-on PER treatment. Multivariate Cox regression analysis showed that only ESES duration before PER administration was a risk factor for PER treatment failure, and the other factors had no effect on the therapeutic effect.

**Conclusion:** PER add-on treatment has a good therapeutic effect on ESES and can be used as an alternative to corticosteroid and benzodiazepines. The therapeutic effect of PER add-on treatment was not related to the dose. A longer ESES duration results in a worse therapeutic effect. Therefore, more aggressive treatment measures should be implemented for ESES.

## 1 Introduction

Electrical status epilepticus during sleep (ESES) is a special electroencephalogram (EEG) phenomenon that refers to a sleep-induced, non-rapid eye movement sleep (NREM) state of continuous or near-continuous emission of 1.5- to 3-Hz spikes and slow waves (Galanopoulou et al., 2000). ESES shows an interictal rather than an ictal EEG form, and the spike wave index (SWI) of slow-wave sleep (SWS) is an important indicator for diagnosis. However, due to different counting methods, no unified diagnostic standard has been established for ESES. The SWI has been reported to be between 25% and 85% (Altunel et al., 2017; Scholtes et al., 2005; Scheltens-De Boer, 2009). ESES can occur in a variety of epilepsy syndromes in children, including epileptic encephalopathy, such as epilepsy with continuous spikes and waves during slow sleep (CSWS), acquired epileptic aphasia, and Lennox-Gastaut syndrome, and in children with benign epilepsy with central-temporal spikes (BECT), which has recently been renamed as self-limited epilepsy with centrotemporal spikes (SeLECTS), and other self-limited focal epilepsy syndromes (Caraballo et al., 2013; Fejerman et al., 2000; Galanopoulou et al., 2000; Specchio et al., 2022). Although epileptic syndromes with ESES and the ESES phenomenon itself are mostly self-limiting with age, a risk of cortical functional impairment emerges with a prolonged ESES duration, which may affect the long-term psychological and cognitive prognoses of patients. Therefore, ESES treatment still attracts attention (Altunel et al., 2017; Tsuru et al., 2000; Veggiotti et al., 2012).

Due to differences in inclusion criteria, epidemiological data on ESES are limited, but the number of patients with ESES is generally believed to be approximately 0.2%–1.0% of the total number of children with epilepsy (Nickels and Wirrell, 2008; Sanchez Fernandez et al., 2012; Veggiotti et al., 2012). Although the incidence of ESES is relatively low, its treatment is often difficult. Even if epileptic seizures have been completely controlled, ESES can persist (Sánchez Fernández et al., 2015). Currently, no recognized guideline recommendation is available for the treatment of ESES, and antiepileptic drug therapy based on comprehensive consideration of the type of seizures and the diagnosis of epilepsy syndrome is advocated (Veggiotti et al., 2016; Wiwattanadittakul et al., 2020). At present, many small-sample studies have reported that various commonly used antiepileptic drugs have poor therapeutic effects on ESES, and phenytoin, carbamazepine, and oxcarbazepine may even aggravate the risk of ESES (Inutsuka et al., 2006; Kramer et al., 2009; Pavlidis et al., 2015). Pulse corticosteroid therapy and high-dose benzodiazepine therapy are considered effective for ESES, but significant adverse reactions limit their long-term application (Kramer et al., 2009; Sanchez Fernandez et al., 2013).

Perampanel (PER), a novel antiepileptic drug, is a noncompetitive antagonist that selectively acts on α-amino-3-hydroxy-5-methyl-4-isoxazolepropionic acid (AMPA) receptors. It was approved in China in September 2019 for add-on treatment of focal epilepsy (with or without secondary generalized seizures) at 12 years of age and older and in July 2021 for monotherapy for focal epilepsy (with or without secondary generalized seizures) at age 4 years of age and older. The efficacy and tolerability of PER in the treatment of children and adults with epilepsy have been demonstrated in the previous studies (Fernandes et al., 2021; Operto et al., 2021; Trinka et al., 2016). At present, many published reports indicate that PER has good efficacy in treating a variety of focal epilepsy syndromes and refractory epilepsy in children, and its safety and tolerability are good (Chinvarun et al., 2021; Gil-Nagel et al., 2018; Youn et al., 2018). It also showed that PER did not negatively affect the cognitive and executive functions of children with epilepsy (Operto et al., 2021). However, the therapeutic effect of PER on ESES has not been reported. At the same time, considering that GABAergic and glutamatergic neuronal circuit abnormalities jointly participate in the pathophysiological basis of ESES (Sanchez Fernandez et al., 2012), while PER, as a noncompetitive antagonist of glutamate AMPA receptor, can inhibit glutamatergic neuronal excitatory transmission, we speculate that it may also have a therapeutic effect on ESES. In this study, we reviewed SWI improvement in children with focal epilepsy and ESES who were treated in the Epilepsy Center of Shengjing Hospital of China Medical University after 6 months of add-on treatment with PER and analyzed the possible influencing factors of PER’s efficacy to provide a new alternative for ESES treatment.

## 2 Materials and methods

### 2.1 Patients and study design

This is a single-center retrospective observational study designed to observe the effectiveness and safety of PER in pediatric patients with epilepsy and ESES. All data were collected from pediatric patients with focal epilepsy and ESES who were treated at the Epilepsy Center of Shengjing Hospital of China Medical University between January 2016 and March 2022. The inclusion criteria were as follows: 1) age 4–16 years; 2) SWI ≥ 25%; 3) PER add-on treatment with a PER treatment time ≥ 24 weeks at the last follow-up; and 4) long-range EEG repeated every 12–24 weeks after PER use and available data for calculation of the SWI. The exclusion criteria were as follows: 1) a lack of long-range EEG data and 2) Lennox-Gastaut syndrome or other secondary epilepsy.

The primary efficacy endpoint was the proportion of patients with ESES resolution after 24 weeks of PER add-on treatment (defined as the response rate). The secondary efficacy endpoints included the proportion of patients with ESES resolution at the last follow-up (defined as the overall response rate), the median time to ESES resolution, and the estimated probability of ESES resolution at 24 weeks of PER treatment (defined as the resolution rate). The resolution of ESES was defined as SWI < 20%. The safety endpoint was the proportion of patients with adverse events (AEs) during PER add-on treatment. AEs were collected from clinical records, and only AEs considered by the investigator to be related to PER were included in the analysis.

### 2.2 Perampanel medication method

All pediatric patients received PER add-on treatment. The initial dose was 1 or 2 mg according to body weight (<30 kg or ≥ 30 kg), and the dose was increased by 1 or 2 mg every 1–2 weeks. The PER dosage was individually adjusted to a maximum of 12 mg based on the child’s response and tolerability, and the maximum dose should not exceed 12 mg.

### 2.3 Data collection

#### 2.3.1 Spike wave index calculation and electroencephalogram interpretation

EEG was recorded using a Nihon-Kohden video-EEG (Tokyo, Japan) with an 18-channel, and the international 10–20 system was used for electrode placement. The duration of each EEG monitoring session for all patients was at least 4 h, including at least 1 complete sleep cycle. SWI = the number of seconds of spikes and slow waves in the NREM phase during EEG monitoring ÷ the total number of seconds in NREM × 100%. Based on the median baseline SWI of all enrolled pediatric patients, the pediatric patients were further divided into the group of ESES with SWI ≥ 60% and the group of ESES with SWI < 60%. The therapeutic effect on ESES was divided into complete resolution and non-resolution. The response rate was determined from the proportion of patients with complete resolution of ESES.

#### 2.3.2 Other data collected

Other collected data included sex, age, past medical history (including birth history and perinatal conditions), family history, age at epilepsy diagnosis, age at ESES diagnosis, ESES duration before add-on PER treatment, medications before PER, whether epileptic seizures were still present before add-on PER treatment, head magnetic resonance imaging (MRI) results, cognitive function before add-on PER treatment (cognitive function was assessed using a scale suitable for the patient’s age or observational information from parents and teachers), whether first-line treatment was performed before add-on PER treatment (first-line treatment was defined as corticosteroid and/or high-dose benzodiazepines), epileptic seizure history, and the SWI after add-on PER treatment.

### 2.4 Statistical analysis

Statistical analysis was performed using IBM SPSS 20.0 software (IBM Corp., Armonk, NY, United States). The mean and standard deviation were used to represent numerical variables, and frequencies and percentages were used to represent categorical variables. Survival analysis was performed using ESES resolution after add-on PER treatment as the outcome variable. The Kaplan‒Meier method was used to calculate the probability of ESES resolution and the median time to ESES resolution. We did exploratory univariate and multivariate analysis of several factors that may affect the resolution/non-resolution of ESES using log-rank test and Cox regression, separately. Factors for univariate and multivariate analysis included sex, age at seizure onset, age at ESES diagnosis, ESES duration, age at the time of add-on PER initiation, cognitive behavioral abnormalities, MRI abnormalities, awake EEG discharge (unilateral or bilateral), first-line treatment before add-on PER initiation, the number of basic antiepileptic drugs, the duration of antiepileptic drug treatment at baseline, baseline SWI, baseline seizure status, and PER dose. *p* < 0.05 was considered statistically significant.

## 3 Results

### 3.1 Patient demographic information and disease conditions

According to medical records, a total of 54 patients met the inclusion criteria, including 33 males and 21 females. The mean age at ESES diagnosis was 7.58 ± 2.40 years. The mean age at add-on PER initiation was 9.69 ± 2.12 years. The mean ESES duration before add-on PER initiation was 25.31 ± 15.12 months. Among the 54 patients, 13 patients had mild MRI abnormalities, 6 of whom had small softening lesions distributed in the forehead, lateral ventricle, posterior horn of the lateral ventricle, or occipital region. Three cases of hippocampal asymmetry, 2 cases of nonspecific demyelination changes in the posterior horn of the lateral ventricle, 1 case of mild hippocampal sclerosis and 1 case of left small choroidal fissure cyst were noted. Among the 54 children, 26 had cognitive behavioral abnormalities before add-on PER initiation. Among them, 11 patients were identified by parents and teachers with learning difficulties, 8 patients were diagnosed with attention deficit hyperactivity disorder (ADHD) after evaluation, 4 patients had global developmental delay, and 3 patients had language developmental disorders.

Forty-nine patients had no seizures when PER was added, and 5 patients still had seizures (4 cases of focal seizures and 1 case of focal seizures evolving into generalized seizures). The EEGs of the 54 patients all showed interictal paroxysms in the awake phase, 27 of which showed unilateral activity (mainly distributed in the central area, temporal area, and occipital area), while 27 EEGs showed bilateral activity. Before PER add-on treatment, 30 patients had an SWI ≥ 60%, and 24 patients had an SWI < 60% (8 of whom had an SWI < 50%). Before add-on PER treatment, 8 patients received oral administration of 1 antiepileptic drug (levetiracetam), 35 patients received oral administration of 2 antiepileptic drugs (28 cases with levetiracetam + valproic acid and 7 cases with levetiracetam + clonazepam), and 11 patients received oral administration of 3 types of antiepileptic drugs (6 cases with levetiracetam + valproic acid + clonazepam and 5 cases with levetiracetam + valproic acid + topiramate). Before add-on PER initiation, 29 patients had been treated with pulse corticosteroid or high-dose benzodiazepines. The baseline demographic and disease characteristics of the patients are shown in Table 1.

**TABLE 1:** Demographics and clinical characteristics.

Characteristics	Total (*n* = 54)
Sex (male, %)	33 (61.1%)
Age at epilepsy diagnosis (mean ± SD, y)	6.41 ± 2.14
Age at ESES diagnosis (mean ± SD, y)	7.58 ± 2.40
Age at add-on PER initiation (mean ± SD, y)	9.69 ± 2.12
The ESES duration before add-on PER initiation (mean ± SD, m)	25.31 ± 15.12
Duration of antiepileptic drug therapy (mean ± SD, m)	29.40 ± 15.67
MRI abnormalities (n, %)	13 (24.1%)
Cognitive behavioral abnormalities (n, %)	26 (48.1%)
Seizures before add-on PER initiation (n, %)	5 (9.3%)
Awake EEG discharge (n, %)	54 (100.0%)
Unilateral	27 (50.0%)
Bilateral	27 (50.0%)
SWI ≥ 60% (n, %)	30 (55.6%)
Numbers of antiepileptic drug combination (n, %)	
1	8 (14.8%)
2	35 (64.8%)
3	11 (20.4%)
Prior first-line therapy	29 (53.7%)

First-line therapy was defined as treatment of pulse corticosteroid or high-dose benzodiazepines.

### 3.2 Perampanel dose and treatment duration

**FIGURE 1:**
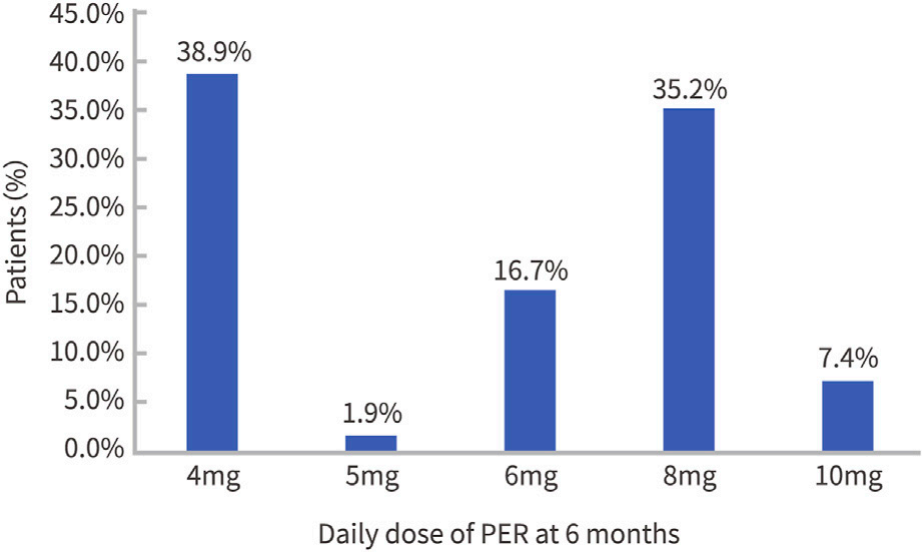
The dose distribution at week 24 of PER add-on treatment.

Among the 54 patients, the mean initial dose of PER was 1.6 ± 4.8 mg, with 1 mg used in 19 cases and 2 mg used in 35 cases. The dose distribution at week 24 of PER add-on treatment is shown in Figure 1. The mean dose was 6.2 ± 2.1 mg. The PER dose in 22 cases was < 6 mg (4 mg: *n* = 21; 5 mg *n* = 1), and the PER dose in 32 cases was ≥ 6 mg (6 mg: *n* = 9, 8 mg: *n* = 19, 10 mg: *n* = 4). The mean duration of PER treatment was 10.3 ± 4.4 months; 10 patients had a PER treatment duration ≥ 12 months, and 2 patients had a PER treatment duration ≥ 24 months.

### 3.3 Effectiveness

After 24 weeks of PER add-on treatment, the ESES of 29 patients resolved, and the total response rate was 53.7%. Among the 25 patients with an SWI that did not resolve at 24 weeks, 9 cases (36%) showed ESES resolution in the subsequent follow-up, with resolution within 8 months to 1 year of PER application in 4 cases, and 5 cases showed resolution within 1–2 years of PER application. Among the 3 pediatric patients whose ESES resolved at 24 weeks and whose follow-up time was longer than 1 year, 1 pediatric patient had recurrence (SWI = 30%), which resolved after 1 month. Overall, at the last follow-up, the ESES resolved in 38 of the 54 patients, and the overall response rate was 70.4%. Among the 29 pediatric patients who did not respond to corticosteroid therapy and benzodiazepine therapy, the response rate was 48.3%. In addition, the 5 children with seizures at the time of add-on PER initiation all reported seizure relief at the 24-week follow-up regardless of ESES resolution (3 cases with resolution, 1 case with an SWI = 30%, and 1 case with an SWI = 50%).

**FIGURE 2:**
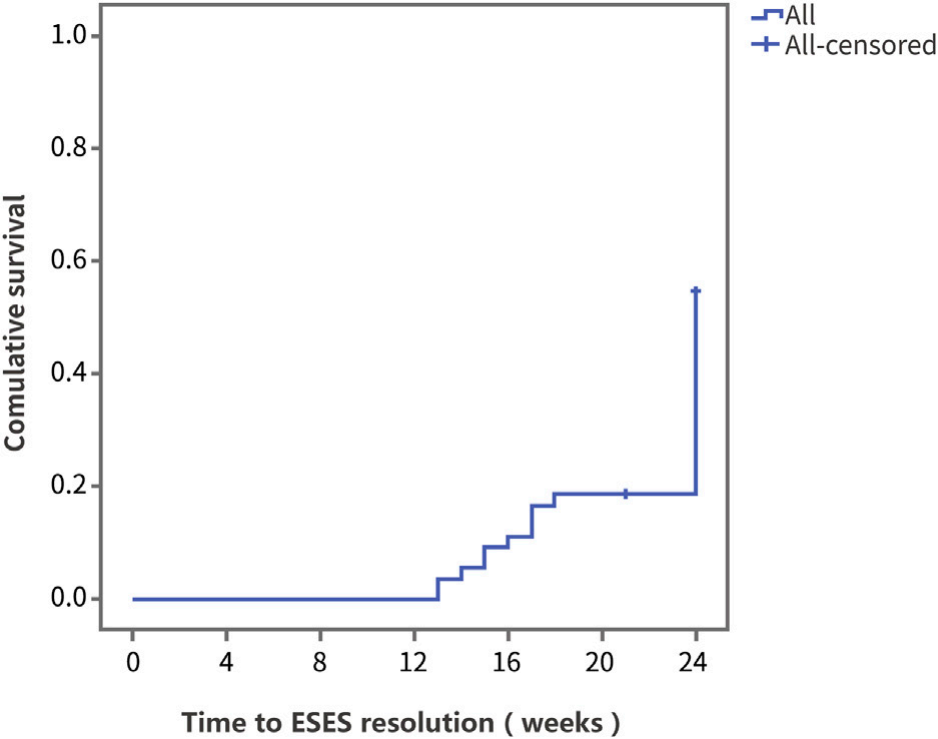
The Kaplan‒Meier curve of the overall population. Survival analysis was performed using ESES resolution as the outcome variable.

Survival analysis was performed using ESES resolution as the outcome variable. Figure 2 shows the Kaplan‒Meier curve of the overall population. At 24 weeks of PER treatment, the resolution rate was 45.5%, and the median time to ESES resolution was 24 weeks.

#### 3.3.1 Analysis of factors influencing the therapeutic effect

**FIGURE 3:**
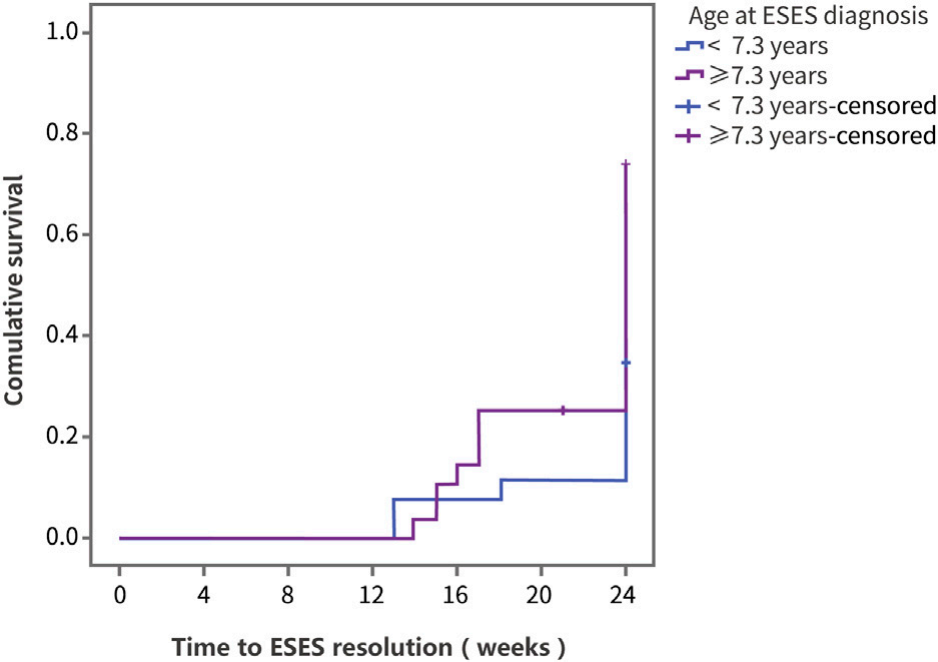
The Kaplan‒Meier curve of the patients with different age at diagnosis. Survival analysis was performed using ESES resolution as the outcome variable.

**FIGURE 4:**
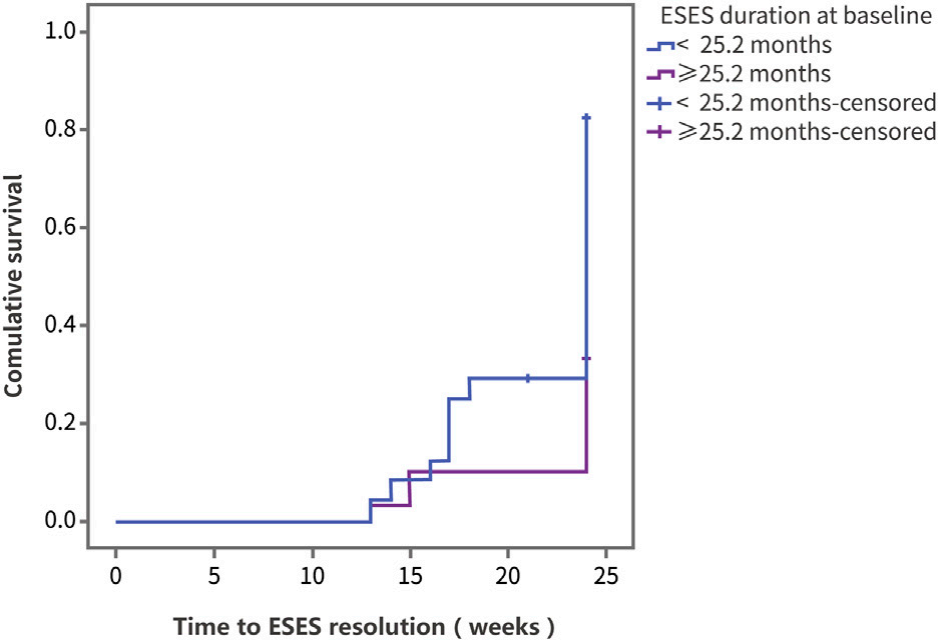
The Kaplan‒Meier curve of the patients with different disease duration. Survival analysis was performed using ESES resolution as the outcome variable.

Using ESES resolution as the outcome variable, the log-rank test was performed on factors including sex, age, disease course, previous treatment, and dose (Supplementary Table S1). In the group of ESES with SWI ≥ 60%, the resolution rate was 44.5% (Supplementary Table S1). The results showed that differences in the therapeutic effect of PER were statistically significant according to the age at ESES diagnosis and the ESES duration before add-on PER treatment. Compared with patients diagnosed with ESES at < 7.3 years of age, patients diagnosed with ESES at ≥ 7.3 years of age had a higher resolution rate (73.8% vs. 34.6%, *p* = 0.008) (Figure 3). For patients with an ESES disease duration ≥ 25.2 months, the resolution rate was higher than that for patients with an ESES disease duration < 25.2 months (82.3% vs. 33.3%, *p* = 0.001) (Figure 4). The results of multivariate Cox regression analysis showed that an ESES duration ≥ 25.2 months before add-on PER initiation was a risk factor for PER treatment failure (hazard ratio (HR) = 0.319, 95% confidence interval (CI): 0.106–0.957, *p* = 0.041). The effects of the remaining factors on the therapeutic effect were not statistically significant (Supplementary Table S2).

### 3.4 Safety

Among the 54 pediatric patients who received PER add-on treatment, 9 patients (16.7%) had at least 1 adverse reaction. Among them, drowsiness occurred in 3 patients (5.6%), and headache occurred in 1 patient (1.9%); all cases appeared in the first 2 weeks after drug initiation and gradually resolved without dose adjustments. Five patients (9.3%) showed signs of impulsivity, irritability, and inattention, with 3 patients showing such signs when the dose was increased to 10 mg and 2 patients showing these signs when the dose was increased to 8 mg, which resolved after the doses were returned to 8 and 6 mg, respectively.

## 4 Discussion

The ESES phenomenon was first described by Patry et al. (1971) in 1971. Although more than 50 years have passed, the concept of ESES is still not unified. At present, some scholars equate ESES with CSWS (Fernandez et al., 2013). However, to avoid confusion, our study emphasized ESES as an EEG phenomenon rather than a specific epilepsy syndrome. The SWI is an important indicator for ESES diagnosis. Although all published studies report the use of the percentage of seconds of spikes and slow waves in the NREM phase, the specific counting methods are not the same (Aeby et al., 2005; Bolsterli et al., 2017; Inutsuka et al., 2006). Some studies have counted the SWI of each complete NREM cycle during EEG recording, while others have counted the first 30 min of the first and last NREM cycles. Some scholars believed that the first 5 min of the first NREM cycle had the highest SWI, which gradually decreased in the subsequent sleep cycle; therefore, only the first 5 min of the first NREM cycle were counted (Öztoprak et al., 2021). Our study used the method of counting each complete NREM cycle and referred to previous studies (van Hirtum-Das et al., 2006), with an SWI ≥ 25% as the diagnostic criteria.

At present, the etiology of ESES is not fully understood, and factors such as brain structural abnormalities, chromosomal/gene abnormalities, inflammation, and immunity caused by congenital and acquired factors may all lead to its occurrence (Kevelam et al., 2012; Siniatchkin et al., 2010; van den Munckhof et al., 2016). However, abnormal activation (depolarization) and silencing (hyperpolarization) of GABAergic neurons in the thalamic reticular nucleus and glutamatergic neurons in the dorsal thalamic nucleus and cerebral cortex are considered to constitute the common pathophysiological basis (Sanchez Fernandez et al., 2012; Bolsterli et al., 2017). Under physiological conditions, the inhibitory GABAergic neurons in this circuit hyperpolarize glutamatergic neurons and cause subsequent rebound depolarization, and the depolarized glutamatergic neurons activate GABAergic neurons again by feedback, thus forming an oscillatory circuit, which is the basis for the formation of sleep spindles, while the ESES phenomenon is considered to be the result of sleep spindle inhibition after the balance is disrupted (Sanchez Fernandez et al., 2012). For the commonly used antiepileptic drugs that are effective for ESES reported in the literature to date (Aeby et al., 2005; Inutsuka et al., 2006; Sanchez Fernandez et al., 2013), including benzodiazepines, valproic acid, ethosuximide, and levetiracetam, the pharmacological mechanisms all involve enhanced GABA receptor-mediated inhibition. As a noncompetitive antagonist of AMPA ionotropic glutamate receptors, PER inhibits glutamate neuronal transmission. Therefore, we speculated that PER may also have therapeutic effects on ESES.

In a meta-analysis published in 2015 by van den Munckhof et al. (2015), corticosteroid therapy was found to have the highest response rate of higher than 80%. Chen et al. (2014) also reported similar results. However, more than 50% of the patients relapsed after 1 year of follow-up. Moreover, long-term corticosteroid therapy may have serious adverse reactions; therefore, it is more suitable for pulse therapy. Among the commonly used antiepileptic drugs, benzodiazepines have the best effect, with a response rate of between 40% and 60%. Adverse responses primarily include drowsiness, decreased muscle tone, and mood changes, which are mostly tolerated (Inutsuka et al., 2006; Sanchez Fernandez et al., 2013; van den Munckhof et al., 2015). Other antiepileptic drugs, such as valproic acid, levetiracetam, and ethosuximide, have also demonstrated therapeutic effects in some small-sample studies, but the overall response rate is less than 50%, and some studies have concluded that these drugs are ineffective (Hughes, 2011; Striano and Capovilla, 2013; Veggiotti et al., 2016; van den Munckhof et al., 2016).

In our retrospective study, we observed for the first time that the response rate of PER add-on treatment for ESES for 24 weeks was 53.7%, which was close to that of benzodiazepines and indicated a good therapeutic effect. Moreover, the response rate among pediatric patients who did not respond to corticosteroid therapy and benzodiazepine therapy was 48.3%, indicating that PER and corticosteroid or benzodiazepine drugs may have different mechanisms of action, which may be used as an alternative to corticosteroid or benzodiazepine drugs. According to the results of the survival analysis, although the probability of ESES resolution of children diagnosed with ESES at ≥ 7.3 years of age was higher than that of children diagnosed with ESES at < 7.3 years of age after PER add-on treatment (73.8% vs. 34.6%, *p* = 0.008), no statistically significant difference in the therapeutic effect was found (64.3% vs. 43.6%, *p* = 0.166) when the patients were grouped according to the age of add-on PER initiation (≥9.8 years vs. < 9.8 years), indicating that an earlier age at disease onset corresponds to a worse response to the drug, but not due to older children are more likely to have self-limited ESES. Unlike previous studies (Öztoprak et al., 2021; van den Munckhof et al., 2015), MRI abnormalities did not lead to differences in the treatment results, which may be related to the milder MRI changes in our enrolled children. Cox multivariate regression results showed that an ESES duration > 2 years before add-on PER initiation was a risk factor for treatment failure, which is also consistent with previous reports demonstrating that prolonged discharge affects synaptic plasticity and causes abnormal neural circuit formation, which indicates that ESES may require more aggressive treatment. The effects of the initial dose of PER (1 vs. 2 mg) and the maintenance dose at 6 months (<6 vs. ≥ 6 mg) on efficacy were not statistically significant, which is also consistent with the characteristics of PER, with its therapeutic effect being independent of the plasma concentration in previous observational studies (Steinhoff et al., 2019), suggesting that a lower effective dose can be selected during the treatment process to reduce the risk of adverse reactions.

There are several strengths in our study. It was one of the first to explore the therapeutic effect of PER in the treatment of pediatric patients with focal epilepsy and ESES. Factors influencing the therapeutic effect have been analyzed in the study as well. We believe that our study plays an essential, instructive role in clinical practice of PER treatment. However, our study used a single-center retrospective approach, which also has the following limitations. First, the sample size was small, which may cause bias in the results. Second, in this retrospective study, the follow-up interval and follow-up duration of all patients were not fixed, causing us to use data of PER add-on treatment for 6 months to determine the effectiveness, but we could not determine the effectiveness and recurrence rate within a longer treatment period. In addition, because our study included 8 pediatric patients with an SWI between 25% and 50%, we did not divide the treatment effect into resolution, effective (50% reduction in SWI), and ineffective as in other studies. Instead, the effect was simply divided into 2 groups of resolution and non- resolution, which also resulted in 3 cases showing potential effectiveness (2 cases with a decrease from 80% to 30% and 1 case with a decrease from 50% to 25%) being assigned to the non- resolution group. Finally, due to the lack of data, we could not determine improvements in cognitive behavior in children with ESES and cognitive behavioral disorders after PER treatment.

## 5 Conclusion

In summary, through a single-center retrospective study, we found that PER add-on therapy may have a good therapeutic effect on ESES, and the effectiveness was not related to whether corticosteroid and benzodiazepine treatment was performed. The effect of PER add-on treatment is not related to the dose, and a smaller maintenance dose may reduce the probability of adverse reactions. A longer ESES duration results in a worse therapeutic effect; thus, more aggressive treatment measures should be implemented for ESES. This study is the first to focus on the effectiveness of PER in ESES. The above findings need to be validated in a large-scale prospective clinical trial.

## Data Availability

The raw data supporting the conclusion of this article will be made available by the authors, without undue reservation.

## References

[B1] Aeby A. Poznanski N. Verheulpen D. Wetzburger C. van Bogaert P. (2005). Levetiracetam efficacy in epileptic syndromes with continuous spikes and waves during slow sleep: Experience in 12 cases. Epilepsia 46 (12), 1937–1942. 10.1111/j.1528-1167.2005.00337.x 16393159

[B2] Altunel A. Altunel E. O. Sever A. (2017). Response to adrenocorticotropic in attention deficit hyperactivity disorder-like symptoms in electrical status epilepticus in sleep syndrome is related to electroencephalographic improvement: A retrospective study. Epilepsy Behav. 74, 161–166. 10.1016/j.yebeh.2017.06.019 28778058

[B3] Bolsterli B. K. Gardella E. Pavlidis E. Wehrle F. M. Tassinari C. A. Huber R. (2017). Remission of encephalopathy with status epilepticus (ESES) during sleep renormalizes regulation of slow wave sleep. Epilepsia 58 (11), 1892–1901. 10.1111/epi.13910 28960278

[B4] Caraballo R. H. Veggiotti P. Kaltenmeier M. C. Piazza E. Gamboni B. Lopez Avaria M. F. (2013). Encephalopathy with status epilepticus during sleep or continuous spikes and waves during slow sleep syndrome: A multicenter, long-term follow-up study of 117 patients. Epilepsy Res. 105 (1-2), 164–173. 10.1016/j.eplepsyres.2013.02.010 23507330

[B5] Chen J. Yang Z. Liu X. Ji T. Y. Fu N. Wu Y. (2014). Efficacy of methylprednisolone therapy for electrical status epilepticus during sleep in children. Zhonghua Er Ke Za Zhi 52 (9), 678–682. 10.3760/cma.j.issn.0578-1310.2014.09.008 25476430

[B6] Chinvarun Y. Huang C. W. Wu Y. Lee H. F. Likasitwattanakul S. Ding J. (2021). Optimal use of perampanel in asian patients with epilepsy: Expert opinion. Ther. Clin. Risk Manag. 17, 739–746. 10.2147/TCRM.S316476 34321883 PMC8312314

[B7] Fejerman N. Caraballo R. Tenembaum S. N. (2000). Atypical evolutions of benign localization-related epilepsies in children: Are they predictable? Epilepsia 41 (4), 380–390. 10.1111/j.1528-1157.2000.tb00177.x 10756401

[B8] Fernandes M. Dainese F. Operto F. Lattanzi S. Matricardi S. Renna R. (2021). Perampanel effectiveness and tolerability in patients with epilepsy at long-term follow-up. Epilepsy Behav. 121, 108069. 10.1016/j.yebeh.2021.108069 34077902

[B9] Fernandez I. S. Chapman K. E. Peters J. M. Kothare S. V. Nordli D. R. Jr Jensen F. E. (2013). The tower of babel: Survey on concepts and terminology in electrical status epilepticus in sleep and continuous spikes and waves during sleep in north America. Epilepsia 54 (4), 741–750. 10.1111/epi.12039 23163318 PMC5030106

[B10] Galanopoulou A. S. Bojko A. Lado F. Moshé S. L. (2000). The spectrum of neuropsychiatric abnormalities associated with electrical status epilepticus in sleep. Brain Dev. 22 (5), 279–295. 10.1016/s0387-7604(00)00127-3 10891635

[B11] Gil-Nagel A. Burd S. Toledo M. Sander J. W. Lebedeva A. Patten A. (2018). A retrospective, multicentre study of perampanel given as monotherapy in routine clinical care in people with epilepsy. Seizure 54, 61–66. 10.1016/j.seizure.2017.10.015 29288911

[B12] Hughes J. R. (2011). A review of the relationships between Landau-Kleffner syndrome, electrical status epilepticus during sleep, and continuous spike-waves during sleep. Epilepsy Behav. 20 (2), 247–253. 10.1016/j.yebeh.2010.10.015 21242107

[B13] Inutsuka M. Kobayashi K. Oka M. Hattori J. Ohtsuka Y. (2006). Treatment of epilepsy with electrical status epilepticus during slow sleep and its related disorders. Brain Dev. 28 (5), 281–286. 10.1016/j.braindev.2005.09.004 16376508

[B14] Kevelam S. H. Jansen F. E. Binsbergen E. Braun K. P. Verbeek N. E. Lindhout D. (2012). Copy number variations in patients with electrical status epilepticus in sleep. J. Child. Neurol. 27 (2), 178–182. 10.1177/0883073811416006 21954431

[B15] Kramer U. Sagi L. Goldberg-Stern H. Zelnik N. Nissenkorn A. Ben-Zeev B. (2009). Clinical spectrum and medical treatment of children with electrical status epilepticus in sleep (ESES). Epilepsia 50 (6), 1517–1524. 10.1111/j.1528-1167.2008.01891.x 19054417

[B16] Nickels K. Wirrell E. (2008). Electrical status epilepticus in sleep. Semin. Pediatr. Neurol. 15 (2), 50–60. 10.1016/j.spen.2008.03.002 18555191

[B17] Operto F. F. Vivenzio V. Scuoppo C. Padovano C. Roccella M. Quatrosi G. (2021). Perampanel and visuospatial skills in children with epilepsy. Front. Neurol. 12, 696946. 10.3389/fneur.2021.696946 34305800 PMC8296464

[B18] Öztoprak Ü. Köken Ö Y. Aksoy E. Yüksel D. (2021). Spike-wave index assessment and electro-clinical correlation in patients with encephalopathy associated with epileptic state during slow sleep (ESES/CSWS); single-center experience. Epilepsy Res. 170, 106549. 10.1016/j.eplepsyres.2021.106549 33450525

[B19] Patry G. Lyagoubi S. Tassinari C. A. (1971). Subclinical "electrical status epilepticus" induced by sleep in children. A clinical and electroencephalographic study of six cases. Arch. Neurol. 24 (3), 242–252. 10.1001/archneur.1971.00480330070006 5101616

[B20] Pavlidis E. Rubboli G. Nikanorova M. Kölmel M. S. Gardella E. (2015). Encephalopathy with status epilepticus during sleep (ESES) induced by oxcarbazepine in idiopathic focal epilepsy in childhood. Funct. Neurol. 30 (2), 139–141. 10.11138/fneur/2015.30.2.139 26415787 PMC4610762

[B21] Sánchez Fernández I. Loddenkemper T. Galanopoulou A. S. Moshé S. L. (2015). Should epileptiform discharges be treated? Epilepsia 56 (10), 1492–1504. 10.1111/epi.13108 26293670 PMC6294573

[B22] Sanchez Fernandez I. Loddenkemper T. Peters J. M. Kothare S. V. (2012). Electrical status epilepticus in sleep: Clinical presentation and pathophysiology. Pediatr. Neurol. 47 (6), 390–410. 10.1016/j.pediatrneurol.2012.06.016 23127259

[B23] Sanchez Fernandez I. Peters J. M. An S. Bergin A. M. Takeoka M. Rotenberg A. (2013). Long-term response to high-dose diazepam treatment in continuous spikes and waves during sleep. Pediatr. Neurol. 49 (3), 163–170. e4. 10.1016/j.pediatrneurol.2013.04.027 23953953 PMC6382391

[B24] Scheltens-De Boer M. (2009). Guidelines for EEG in encephalopathy related to ESES/CSWS in children. Epilepsia 50 (7), 13–17. 10.1111/j.1528-1167.2009.02211.x 19682043

[B25] Scholtes F. B. Hendriks M. P. Renier W. O. (2005). Cognitive deterioration and electrical status epilepticus during slow sleep. Epilepsy Behav. 6 (2), 167–173. 10.1016/j.yebeh.2004.11.001 15710299

[B26] Siniatchkin M. Groening K. Moehring J. Moeller F. Boor R. Brodbeck V. (2010). Neuronal networks in children with continuous spikes and waves during slow sleep. Brain 133 (9), 2798–2813. 10.1093/brain/awq183 20688812

[B27] Specchio N. Wirrell E. C. Scheffer I. E. Nabbout R. Riney K. Samia P. (2022). International league against epilepsy classification and definition of epilepsy syndromes with onset in childhood: Position paper by the ILAE task force on nosology and definitions. Epilepsia 63 (6), 1398–1442. 10.1111/epi.17241 35503717

[B28] Steinhoff B. J. Hübers E. Kurth C. Jürges Kehl-Kork U. (2019). Plasma concentration and clinical effects of perampanel-The Kork experience. Seizure 67, 18–22. 10.1016/j.seizure.2019.02.022 30852267

[B29] Striano P. Capovilla G. (2013). Epileptic encephalopathy with continuous spikes and waves during sleep. Curr. Neurol. Neurosci. Rep. 13 (7), 360. 10.1007/s11910-013-0360-5 23666433

[B30] Trinka E. Steinhoff B. J. Nikanorova M. Brodie M. J. (2016). Perampanel for focal epilepsy: Insights from early clinical experience. Acta Neurol. Scand. 133 (3), 160–172. 10.1111/ane.12529 26506904 PMC4738453

[B31] Tsuru T. Mori M. Mizuguchi M. Momoi M. Y. (2000). Effects of high-dose intravenous corticosteroid therapy in Landau-Kleffner syndrome. Pediatr. Neurol. 22 (2), 145–147. 10.1016/s0887-8994(99)00127-7 10738922

[B32] van den Munckhof B. De Vries E. E. Braun K. P. Boss H. M. Willemsen M. A. van Royen-Kerkhof A. (2016). Serum inflammatory mediators correlate with disease activity in electrical status epilepticus in sleep (ESES) syndrome. Epilepsia 57 (2), e45–e50. 10.1111/epi.13274 26666401

[B33] van den Munckhof B. van Dee V. Sagi L. Caraballo R. H. Veggiotti P. Liukkonen E. (2015). Treatment of electrical status epilepticus in sleep: A pooled analysis of 575 cases. Epilepsia 56 (11), 1738–1746. 10.1111/epi.13128 26337159

[B34] van Hirtum-Das M. Licht E. A. Koh S. Wu J. Y. Shields W. D. Sankar R. (2006). Children with ESES: Variability in the syndrome. Epilepsy Res. 70 (1), S248–S258. 10.1016/j.eplepsyres.2006.01.020 16806829

[B35] Veggiotti P. Pera M. C. Olivotto S. De Giorgis V. (2016). How to manage electrical status epilepticus in sleep. J. Clin. Neurophysiol. 33 (1), 3–9. 10.1097/WNP.0000000000000235 26840869

[B36] Veggiotti P. Pera M. C. Teutonico F. Brazzo D. Balottin U. Tassinari C. A. (2012). Therapy of encephalopathy with status epilepticus during sleep (ESES/CSWS syndrome): An update. Epileptic Disord. 14 (1), 1–11. 10.1684/epd.2012.0482 22426353

[B37] Wiwattanadittakul N. Depositario-Cabacar D. Zelleke T. G. (2020). Electrical status epilepticus in sleep (ESES) - treatment pattern and EEG outcome in children with very high spike-wave index. Epilepsy Behav. 105, 106965. 10.1016/j.yebeh.2020.106965 32155577

[B38] Youn S. E. Kim S. H. Ko A. Lee S. H. Lee Y. M. Kang H. C. (2018). Adverse events during perampanel adjunctive therapy in intractable epilepsy. J. Clin. Neurol. 14 (3), 296–302. 10.3988/jcn.2018.14.3.296 29971974 PMC6031997

