# Supplementary material for: Effectiveness of perampanel in the treatment of pediatric patients with focal epilepsy and ESES: A single-center retrospective study
Source: Front Pharmacol. 2022 Oct 7;13:1026836. doi: 10.3389/fphar.2022.1026836 (PMC9585220; doi:10.3389/fphar.2022.1026836)
Supplement: Supplementary file 1 [file Table1.DOCX]

Supplementary Material

# Supplementary tables

**Table 1. The result of univariate analysis using the log-rank test**

|  |  |  |  | **Time to SWI-free (weeks)** | |  |  |
| --- | --- | --- | --- | --- | --- | --- | --- |
|  |  | **n** | **Resolution rate at 24 weeks (%)** | **mean** | **95% confidence interval** | **Log-rank Chi-squared** | ***P*** |
| Sex | Male  Female | 33  21 | 49.6  61.9 | 22.18  22.81 | (20.92,23.45)  (21.48,24.14) | 0.235 | 0.628 |
| Age | < 9.8  ≥ 9.8 | 26  28 | 43.6  64.3 | 22.65  22.21 | (21.33,23.98)  (20.90,23.53) | 1.919 | 0.166 |
| Age at seizure onset | < 6.5  ≥ 6.5 | 26  28 | 43.7  64.3 | 22.92  21.96 | (21.68,24.17)  (20.60,23.33) | 2.527 | 0.112 |
| Age at ESES diagnosis | < 7.3  ≥ 7.3 | 26  28 | 34.6  73.8 | 22.92  21.96 | (21.66,24.18)  (20.61,23.32) | 7.118 | **0.008*** |
| ESES duration before enrollment | < 25.2  ≥ 25.2 | 24  30 | 82.3  33.3 | 21.67  23.03 | (20.13,23.21)  (21.93,24.13) | 11.608 | **0.001*** |
| Cognitive dysfunction before enrollment | No  Yes | 28  26 | 55.1  53.8 | 22.07  22.81 | (20.62,23.52)  (21.67,23.94) | 0.102 | 0.749 |
| MRI abnormalities | No  Yes | 41  13 | 56.1  49.2 | 22.27  22.92 | (21.14,23.39)  (21.42,24.43) | 0.254 | 0.615 |
| EEG background | Unilateral  Bilateral | 27  27 | 59.3  49.7 | 21.96  22.89 | (20.45,23.48)  (21.83,23.95) | 0.719 | 0.396 |
| First-line therapy | No  Yes | 25  29 | 60  49.6 | 22.24  22.59 | (20.78,23.70)  (21.39,23.79) | 0.502 | 0.479 |
| Numbers of drug combination before enrollment | 1  2  3 | 8  35  11 | 87.5  51.4  36.4 | 22.00  22.34  24.00 | (19.38,24.62)  (21.19,23.50)  (20.84,25.16) | 3.813 | 0.282 |
| Duration of antiepileptic treatment before PER add-on initiation | < 40.2  ≥ 40.2 | 27  27 | 66.7  42 | 21.85  23.00 | (20.41,23.30)  (21.86,24.14) | 3.573 | 0.059 |
| SWI at baseline | < 60%  ≥ 60% | 24  30 | 66.7  44.5 | 21.88  22.87 | (20.32,23.43)  (21.76,23.97) | 2.730 | 0.098 |
| Seizure status at baseline | No  Yes | 49  5 | 54  60 | 22.63  20.40 | (21.73,23.54)  (15.48,25.32) | 0.511 | 0.475 |
| Initial dose of PER at baseline | 1  2 | 19  35 | 43.9  60 | 22.74  22.26 | (21.18,24.20)  (21.06,23.46) | 1.074 | 0.300 |
| Last dose of PER | < 6  ≥ 6 | 22  32 | 42  62.5 | 22.14  22.63 | (20.57,23.70)  (21.47,23.78) | 0.908 | 0.341 |

*: *P* < 0.05

**Table 2. The result of multivariate analysis using the Cox regression model**

|  |  |  |  | **95% Confidence Interval** | |
| --- | --- | --- | --- | --- | --- |
|  | ***B*** | ***P*** | ***HR*** | **Lower bound** | **Upper bound** |
| Age at seizure onset | -0.091 | 0.912 | 0.913 | 0.181 | 4.597 |
| Age at ESES diagnosis | 0.604 | 0.379 | 1.83 | 0.476 | 7.032 |
| ESES duration before enrollment | -1.142 | **0.041*** | 0.319 | 0.106 | 0.957 |
| Age at PER add-on initiation | 0.284 | 0.704 | 1.328 | 0.307 | 5.741 |
| Cognitive dysfunction before enrollment | -0.188 | 0.686 | 0.828 | 0.332 | 2.065 |
| MRI abnormalities | -0.244 | 0.630 | 0.784 | 0.291 | 2.114 |
| Awake EEG discharge | 0.541 | 0.336 | 1.718 | 0.571 | 5.172 |
| First-line therapy | 0.294 | 0.547 | 1.342 | 0.516 | 3.494 |
| Numbers of drug combination before enrollment (1) | -0.261 | 0.765 | 0.770 | 0.139 | 4.260 |
| Numbers of drug combination before enrollment (2) | 0.636 | 0.312 | 1.889 | 0.551 | 6.484 |
| Duration of antiepileptic treatment before PER add-on initiation | -1.026 | 0.056 | 0.358 | 0.125 | 1.026 |
| SWI at baseline | -0.851 | 0.115 | 0.427 | 0.148 | 1.232 |
| Seizure status at baseline | -0.232 | 0.768 | 0.793 | 0.170 | 3.707 |
| Initial dose of PER at baseline | 0.369 | 0.459 | 1.447 | 0.544 | 3.845 |
| Dose of PER at 6 months | -0.275 | 0.598 | 0.759 | 0.273 | 2.115 |

*: *P* < 0.05
